# Chemokine-Like Receptor 1 mRNA Weakly Correlates with Non-Alcoholic Steatohepatitis Score in Male but Not Female Individuals

**DOI:** 10.3390/ijms17081335

**Published:** 2016-08-18

**Authors:** Maximilian Neumann, Elisabeth M. Meier, Lisa Rein-Fischboeck, Sabrina Krautbauer, Kristina Eisinger, Charalampos Aslanidis, Rebekka Pohl, Thomas S. Weiss, Christa Buechler

**Affiliations:** 1 Department of Internal Medicine I, Regensburg University Hospital, 93053 Regensburg, Germany; maximilian.neumann@stud.uni-regensburg.de (M.N.); elisabeth.meier@klinik.uni-regensburg.de (E.M.M.); lisa.rein-fischboeck@klinik.uni-regensburg.de (L.R.-F.); sabrina.krautbauer@klinik.uni-regensburg.de (S.K.); kristina.eisinger@gmx.de (K.E.); rebekka.pohl@klinik.uni-regensburg.de (R.P.); 2 Institute of Clinical Chemistry and Laboratory Medicine, Regensburg University Hospital, 93053 Regensburg, Germany; charalampos.aslanidis@klinik.uni-regensburg.de; 3 Children’s University Hospital (KUNO), Regensburg University Hospital, 93053 Regensburg, Germany; thomas.weiss@klinik.uni-regensburg.de

**Keywords:** liver steatosis, fibrosis, gender, type 2 diabetes

## Abstract

The chemokine-like receptor 1 (CMKLR1*)* ligands resolvin E1 and chemerin are known to modulate inflammatory response. The progression of non-alcoholic fatty liver disease (NAFLD) to non-alcoholic steatohepatitis (NASH) is associated with inflammation. Here it was analyzed whether hepatic *CMKLR1* expression is related to histological features of NASH. Therefore, *CMKLR1* mRNA was quantified in liver tissue of 33 patients without NAFLD, 47 patients with borderline NASH and 38 patients with NASH. Hepatic *CMKLR1* mRNA was not associated with gender and body mass index (BMI) in the controls and the whole study group. *CMKLR1* expression was similar in controls and in patients with borderline NASH and NASH. In male patients weak positive correlations with inflammation, fibrosis and NASH score were identified. In females *CMKLR1* was not associated with features of NAFLD. Liver *CMKLR1* mRNA tended to be higher in type 2 diabetes patients of both genders and in hypercholesterolemic women. In summary, this study shows that hepatic *CMKLR1* mRNA is weakly associated with features of NASH in male patients only.

## 1. Introduction

Non-alcoholic fatty liver disease (NAFLD) is a widespread cause of chronic liver injury and its progressive form non-alcoholic steatohepatitis (NASH) is characterized by hepatic inflammation and fibrosis [1,2]. NAFLD is related to the metabolic syndrome, and importantly, these patients have a high prevalence of developing hypertension, type 2 diabetes and dyslipidemia [3]. Chemokine-like receptor 1 (CMKLR1) is expressed by immune cells including subsets of dendritic cells, macrophages and natural killer cells [4,5]. The adipokine chemerin attracts CMKLR1-expressing cells to sites of inflammation [4,5]. Hepatic chemerin levels are changed in NAFLD, suggesting a function of this chemoattractant factor herein [6,7,8]. Importantly, higher chemerin expression has been described in human NASH while levels are unchanged in patients with borderline NASH and even reduced in human fatty liver [6,7,8]. Serum chemerin is increased in obesity and positive associations with serum lipids, blood glucose and blood pressure suggest a function of this chemokine in metabolic diseases [9]. Data on serum chemerin in NAFLD are not concordant and higher as well as normal levels have been described [10].

CMKLR1 also binds the potent anti-inflammatory mediator resolvin E1 which is generated from ω-3 eicosapentaenoic acid [11].

CMKLR1 deficiency in mice is not related to changes in body weight, inflammation, glucose tolerance and dyslipidemia. Importantly, comparable results have been obtained in animals fed a standard chow and mice given a high-fat diet [12]. In a second study, a high-fat, high-cholesterol diet did not differentially affect body weight and insulin resistance in CMKLR1-null mice and the respective control animals. Of note, hepatic inflammation and expression of fibrotic genes is unchanged in the liver of CMKLR1-deficient mice [13]. Nevertheless, reduced body weight and body fat irrespective of low- or high-fat diet has also been reported in CMKLR1 knock-out mice. Despite decreased hepatic and adipose tissue inflammation, these animals have impaired glucose disposal in muscle and fat [14].

CMKLR1 is highly abundant in the liver and is expressed by primary human hepatocytes, hepatic stellate cells, Kupffer cells and bile duct cells [15]. In patients with chronic hepatitis C, liver *CMKLR1* mRNA is, however, not related to the inflammatory activity grade. *CMKLR1* mRNA levels are comparable in males and females and expression is significantly reduced in women with advanced liver fibrosis [16].

In human NASH liver, *CMKLR1* mRNA is even induced and IL-6 is suggested to contribute to *CMKLR1* upregulation [7]. Associations of hepatic *CMKLR1* expression with hepatocyte ballooning, lobular inflammation and fibrosis have not been identified in human NAFLD [7]. The limitation of this study is that only three patients with NASH were enrolled [7]. To our knowledge, gender-specific expression of *CMKLR1* in human NAFLD has not been analyzed so far.

Here, hepatic *CMKLR1* was quantified in a relatively large cohort of patients with histologically proven NAFLD. Analysis was performed for both genders separately to identify possible sex-related differences.

## 2. Results

### 2.1. Hepatic Chemokine-Like Receptor 1 (CMKLR1) mRNA in the Human Liver

Recently, Döcke et al. analyzed *CMKLR1* mRNA in 34 controls, 10 patients with a NASH score of 3–4 (undefined or borderline NASH) and three patients with a score equal or above 5 [7]. Because in that cohort the number of patients with definite NASH was quite small, we decided to determine *CMKLR1* mRNA in a larger study group. *CMKLR1* mRNA was measured in a cohort of 118 patients including 33 controls with normal liver, 47 patients with a NASH score ranging from 1.0 to 4.5 (borderline NASH) and 38 patients with a NASH score equal or above 5 (Table 1).

**Figure 1:**
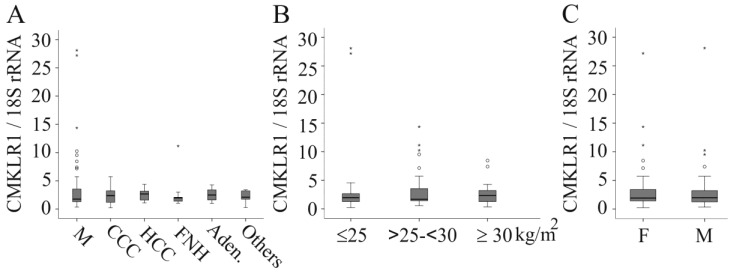
*CMKLR1* mRNA (normalized for 18S rRNA) in the human liver. (**A**) *CMKLR1* mRNA in liver tissues of patients stratified for surgery indications (M, liver metastases of extrahepatic tumours; CCC, cholangiocarcinoma; HCC, hepatocellular carcinoma; FNH, focal nodular hyperplasia of the liver; Aden., adenoma); (**B**) *CMKLR1* mRNA in the liver of normal weight (Body mass index (BMI) ≤ 25 kg/m^2^), overweight (BMI > 25 and < 30 kg/m^2^) and corpulent (BMI ≥ 30 kg/m^2^) patients; (**C**) *CMKLR1* mRNA in female (F) and male (M) liver.

Indications for surgery (hepatocellular carcinoma, adenoma, hepatic metastases of extrahepatic tumours, focal nodular hyperplasia of the liver and cholangiocarcinoma) were not associated with altered *CMKLR1* mRNA levels in the liver tissues used herein (Figure 1A).

In the patients with normal liver and in the whole study group, *CMKLR1* mRNA did not correlate with age (*r* = −0.249, *p* = 0.162 in the control group and *r* = 0.050, *p* = 0.596 in the whole cohort) or Body mass index (BMI) (*r* = −0.189, *p* = 0.292 in the control group and *r* = 0.057, *p* = 0.546 in the whole cohort; data not shown). *CMKLR1* mRNA was similarly expressed in the liver of normal-weight (BMI ≤ 25 kg/m^2^), overweight (BMI > 25 and < 30 kg/m^2^) and obese patients (BMI ≥ 30 kg/m^2^) (Figure 1B). *CMKLR1* mRNA was not related to gender (Figure 1C).

### 2.2. Hepatic CMKLR1 mRNA in Human Non-Alcoholic Fatty Liver Disease (NAFLD)

**Figure 2:**
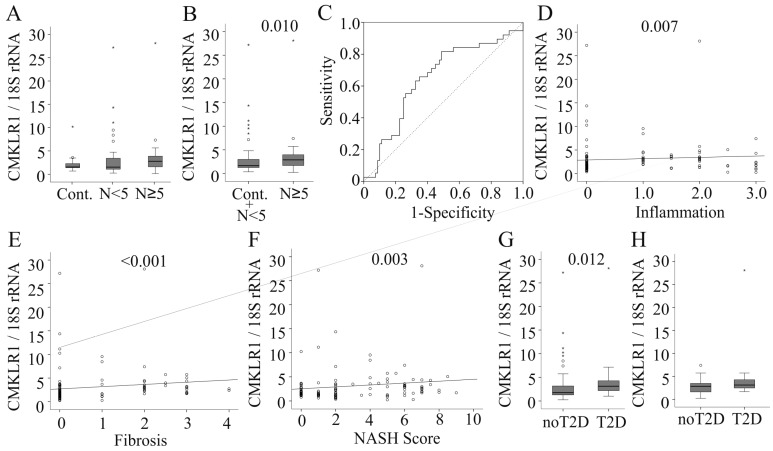
*CMKLR1* mRNA (normalized for 18S rRNA) in non-alcoholic fatty liver disease (NAFLD). (**A**) *CMKLR1* mRNA in liver tissues of patients with healthy liver (Cont.), a non-alcoholic steatohepatitis (NASH) score (*N*) <5 and ≥5; (**B**) *CMKLR1* in controls and patients with a NASH score <5 compared to those with NASH; (**C**) Receiver operating characteristic (ROC) curve analysis; (**D**) Correlation of hepatic *CMKLR1* mRNA with inflammation; (**E**) Correlation of hepatic *CMKLR1* mRNA with fibrosis; (**F**) Correlation of hepatic *CMKLR1* mRNA with the NASH score; (**G**) *CMKLR1* mRNA in patients with and without type 2 diabetes; (**H**) *CMKLR1* mRNA in NASH patients with and without type 2 diabetes. The *p*-values for significant differences/correlations are shown in the figure.

In patients with definite NASH (NASH score ≥ 5), *CMKLR1* mRNA was not significantly increased compared to controls and compared to patients with borderline NASH (NASH score < 5) (Figure 2A). When the first two groups with similar median values of CMKLR1 mRNA were combined, levels were significantly lower compared to NASH patients (Figure 2B). Receiver operating characteristic (ROC) curve analysis (Figure 2C) revealed an area under the curve (AUC) of 0.648 excluding analysis of *CMKLR1* mRNA as a tool for NASH diagnosis. Alanine aminotransferase (*r* = 0.025, *p* = 0.802), aspartate aminotransferase (*r* = −0.79, *p* = 0.447), alkaline phosphatase (*r* = 0.139, *p* = 0.167) and bilirubin (*r* = −0.104, *p* = 0.295) in serum were not associated with hepatic *CMKLR1* mRNA (data not shown). *CMKLR1* did not correlate with steatosis grade (*r* = 0.146, *p* = 0.114) but positively correlated with inflammation (*r* = 0.248, *p* = 0.007), fibrosis (*r* = 0.425, *p* < 0.001) and NASH score (*r* = 0.272, *p* = 0.003) (Figure 2D–F and data not shown).

Type 2 diabetes, hypertension and dyslipidemia are commonly diagnosed in NASH patients [3]. Systemic chemerin positively correlates with low-density lipoprotein cholesterol, insulin resistance and systolic as well as diastolic blood pressure [9]. Whether hepatic CMKLR1 is associated with metabolic diseases such as hypercholesterolemia or type 2 diabetes has not been evaluated to our knowledge so far.

*CMKLR1* mRNA was similar in the 14 patients with and those without hypercholesterolemia (data not shown). In the 45 hypertensive patients, hepatic *CMKLR1* mRNA was not changed (data not shown). *CMKLR1* mRNA was elevated in the 15 patients with type 2 diabetes (Figure 2G). Type 2 diabetes is a risk factor for NAFLD [2] and the NASH score was significantly higher (*p* = 0.001) in this group.

In the NASH group 11 patients had diabetes, and here, *CMKLR1* mRNA was comparable to that of non-diabetic NASH patients (*p* = 0.201, Figure 2H). Similarly, *CMKLR1* mRNA was unchanged in the 10 patients with NASH and hypercholesterolemia (*p* = 0.935) and the 17 hypertensive NASH patients (*p* = 0.367) compared to NASH patients not suffering from these co-morbidities (data not shown).

### 2.3. Hepatic CMKLR1 mRNA in Females

**Figure 3:**
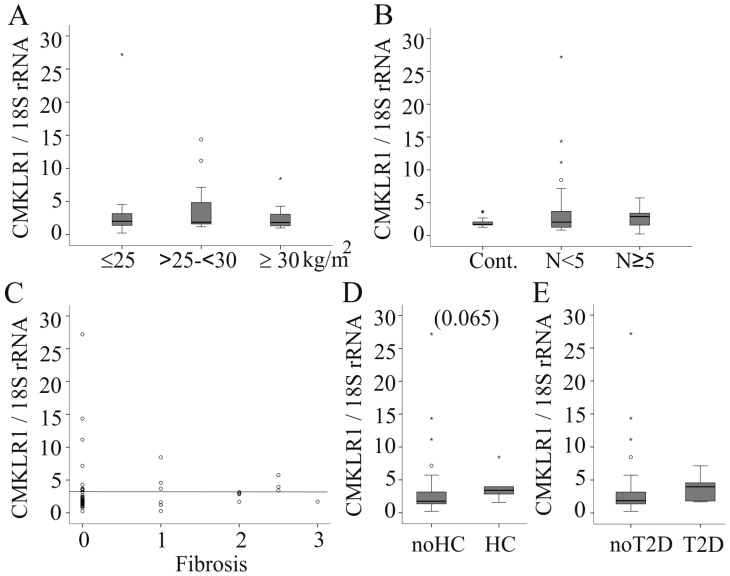
*CMKLR1* mRNA (normalized for 18S rRNA) in female NAFLD patients. (**A**) *CMKLR1* mRNA in the liver of normal-weight (BMI ≤ 25 kg/m^2^), overweight (BMI > 25 and < 30 kg/m^2^) and obese (BMI ≥ 30 kg/m^2^) female patients; (**B**) *CMKLR1* mRNA in liver tissues of female patients with healthy liver (Cont.), a NASH score (*N*) <5 and ≥5; (**C**) Correlation of hepatic *CMKLR1* mRNA in females with fibrosis; (**D**) Hepatic CMKLR1 in females with and without hypercholesterolemia (HC); (**E**) *CMKLR1* mRNA in liver tissues of female patients with and without type 2 diabetes (T2D). Number in brackets indicates a trend.

Recently, gender-specific associations of hepatic *CMKLR1* expression with liver histology have been identified in chronic hepatitis [16]. Therefore, *CMKLR1* mRNA was analyzed in both genders separately. In the female patients 22 had a normal weight, 15 were overweight and 19 were obese. *CMKLR1* mRNA expression was, however, not associated with BMI (*r* = 0.031, *p* = 0.823; Figure 3A). Of the 56 female patients, 17 had normal liver, 25 borderline NASH and 14 NASH. *CMKLR1* mRNA was similarly expressed in the three groups (Figure 3B). There was no difference in the hepatic levels of *CMKLR1* mRNA compared to the combined values of the controls and those with borderline NASH (*p* = 0.609). *CMKLR1* mRNA did not correlate with steatosis grade (*r* = 0.075, *p* = 0.582), inflammation (*r* = 0.054, *p* = 0.693), fibrosis (*r* = 0.248, 0.068) and NASH score (*r* = 0.137, *p* = 0.314) (Figure 3C and data not shown). Alanine aminotransferase (*r* = −0.040, *p* = 0.787), aspartate aminotransferase (*r* = −0.234, *p* = 0.113), alkaline phosphatase (*r* = 0.170, *p* = 0.249), and bilirubin (*r* = −0.066, *p* = 0.647) in serum were not associated with hepatic *CMKLR1* mRNA (data not shown).

*CMKLR1* levels tended to be increased in the six females with hypercholesterinemia (Figure 3D). There was a modest trend to a higher expression in the liver of the five females with type 2 diabetes (*p* = 0.103, Figure 3E). *CMKLR1* expression was not related to hypertension diagnosed in 16 females (data not shown).

### 2.4. Hepatic CMKLR1 mRNA in Males

**Figure 4:**
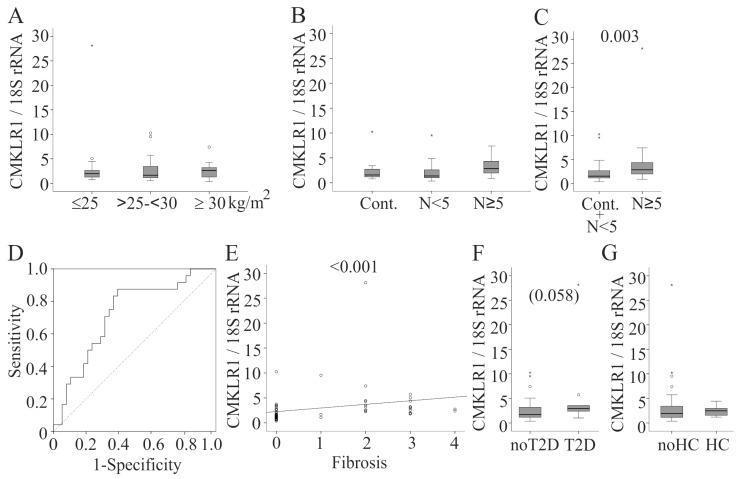
*CMKLR1* mRNA (normalized for 18S rRNA) in male NAFLD patients. (**A**) *CMKLR1* mRNA in the liver of normal-weight (BMI ≤ 25 kg/m^2^), overweight (BMI > 25 and < 30 kg/m^2^) and obese (BMI ≥ 30 kg/m^2^) male patients; (**B**) *CMKLR1* mRNA in liver tissues of male patients with healthy liver (Cont.), a NASH score (*N*) <5 and ≥5; (**C**) *CMKLR1* in male controls and borderline NASH compared to male NASH patients; (**D**) ROC analysis; (**E**) Correlation of hepatic *CMKLR1* mRNA in males with fibrosis; (**F**) Hepatic *CMKLR1* mRNA in males with and without type 2 diabetes (T2D); (**G**) Hepatic *CMKLR1* in males with and without hypercholesterolemia. The *p*-values for significant differences are shown in the figure. Numbers in brackets indicate a trend.

In the male cohort 17 patients had a normal weight, 28 were overweight and 17 were obese. *CMKLR1* mRNA was not associated with BMI (*r* = 0.068, *p* = 0.607; Figure 4A). Of the 62 male patients, 16 had normal liver, 22 borderline NASH and 24 NASH. *CMKLR1* mRNA was similar in the liver of male NASH patients compared to those with borderline NASH and controls. When the last two cohorts were combined, *CMKLR1* mRNA was lower compared to that of NASH patients (Figure 4C). ROC analysis (Figure 4D) revealed an AUC of 0.723. The optimal cut-off point was 1.7 with a sensitivity of 88% and a specificity of 48% to detect NASH. *CMKLR1* mRNA positively correlated with inflammation score (*r* = 0.404, *p* = 0.001), fibrosis score (*r* = 0.555, *p* < 0.001; Figure 4E) and NASH score (*r* = 0.392, *p* = 0.002).

Alanine aminotransferase (*r* = 0.028, *p* = 0.839), aspartate aminotransferase (*r* = 0.055, *p* = 0.705), alkaline phosphatase (*r* = 0.225, *p* = 0.108) and bilirubin (*r* = −0.132, *p* = 0.346) in serum were not associated with hepatic *CMKLR1* mRNA (data not shown).

In males, *CMKLR1* expression tended to be higher in the 10 patients with type 2 diabetes (*p* = 0.058, Figure 4F). Expression was not related to hypertension (29 patients) and hypercholesterinemia (8 patients) (Figure 4G and data not shown).

To exclude that the higher number of type 2 diabetic patients accounts for associations of *CMKLR1* mRNA with NASH in male patients; correlation analysis was performed using data of the 52 males without this co-morbidity. *CMKLR1* mRNA still correlated with inflammation (*r* = 0.378, *p* = 0.006), fibrosis (*r* = 0.547, *p* < 0.001) and NASH score (*r* = 0.312, *p* = 0.024).

## 3. Discussion

We present evidence that hepatic *CMKLR1* mRNA expression is associated with NASH in male patients. Here, positive correlations of *CMKLR1* mRNA with inflammation score, fibrosis score and consequently NASH score have been identified. The correlation coefficients are rather low but associations are highly significant. In females, hepatic *CMKLR1* expression is not related to features of NASH. Although the current study could not identify increased *CMKLR1* expression in human NASH, a recent study reported elevated *CMKLR1* in those patients. Gender-related analysis has not been performed in this cohort [7].

*CMKLR1* mRNA is comparable in both genders in the cohort analyzed herein. Levels of mRNA are also similar in female and male patients with chronic hepatitis C [16]. In hepatitis C patients, *CMKLR1* mRNA is not related to inflammation and a negative association with fibrosis has been identified in females only [16]. *CMKLR1* mRNA regulation in chronic liver disease is therefore influenced by gender and etiology of hepatic injury. The prevalence of NASH is higher in males, and this may be due to the fact that sex hormones affect NASH severity [17,18]. Modest upregulation of *CMKLR1* in male NASH patients obviously contributes an additional factor responsible for gender-related differences in NASH pathology.

Obesity is a risk factor for NAFLD [1]. The mature-onset obesity phenotype has been observed in male but not female CMKLR1-deficient mice [12]. Our results do not show any relation between hepatic *CMKLR1* mRNA and BMI, arguing against an association of liver *CMKLR1* levels and body weight.

Interestingly, hepatic *CMKLR1* tends to be increased in type 2 diabetes patients in both genders and upregulation is significant in the whole cohort. This suggests that elevated *CMKLR1* mRNA in the liver of these patients is not necessarily related to NASH which has a higher prevalence in type 2 diabetes [2]. There is, however, no difference in *CMKLR1* mRNA in male NASH patients with and without type 2 diabetes. Hypercholesterinemia in females is also linked to an increase in hepatic *CMKLR1*. Blockage of cholesterol synthesis in adipocytes does not affect CMKLR1 protein levels, while chemerin is strongly reduced [19]. In hepatocytes, elevation of cellular cholesterol does not change CMKLR1 protein [15]. Therefore, CMKLR1 levels seem not to be related to cellular cholesterol concentrations. Dyslipidemia may nevertheless affect hepatic CMKLR1 activity in females independent of NAFLD. A limitation of the current study is the relatively low number of patients with type 2 diabetes and hypercholesterolemia. There are no patients suffering from these co-morbidities in the control group and only few patients in the borderline NASH group. Therefore, the association of CMKLR1 with type 2 diabetes and/or hypercholesterolemia has to be evaluated in different cohorts using patients who ideally do not suffer from NASH. The main intention of the present study was, however, to identify NAFLD-related changes of this hepatic chemokine receptor. *CMKLR1* mRNA was still associated with inflammation, fibrosis and NASH score when those suffering from type 2 diabetes were excluded.

The association of *CMKLR1* with NASH score in male NASH patients may suggest a higher activity of CMKLR1-related signaling pathways.

Chemerin is abundantly expressed in the liver, and in patients with chronic hepatitis C mRNA levels are similar in males and females [8,16]. In human NASH, hepatic chemerin expression is induced [7] while Deng et al. described lower levels in human fatty liver [6]. Gender-related regulation of liver chemerin has not been evaluated so far. Serum chemerin is found increased in females in some but not all studies [20]. The CMKLR1 receptor is only activated by proteolytic cleaved chemerin [9] and we are unaware of data on gender-related activation of this adipokine. Additional investigations are needed to evaluate whether chemerin signaling is indeed enhanced upon higher expression of hepatic CMKLR1. Further, the physiological and pathophysiological roles of CMKLR1/chemerin signaling in the liver have to still be clarified.

Resolvin E1 is an additional ligand of CMKLR1 [11]. In ob/ob mice, this lipid ameliorates insulin sensitivity and hepatic steatosis [21]. In a murine model of liver fibrosis induced by *Schistosoma japonicum* infection, resolvin E1 treatment reduces the growth of granulomas and thereby delays hepatic fibrogenesis [22]. However, resolvin E1 fails to improve liver injury in mice fed an atherogenic diet to induce NASH [23]. Resolvins are derived from ω-3 polyunsaturated fatty acids [24] and ethyl-eicosapentanoic acid could not ameliorate NASH in a clinical trial [25]. Therefore, the potential beneficial effects of resolvins in NASH have to be proven in future studies.

The non-availability of protein from the respective liver tissues may be considered as a limitation of our study. Recently, our group has shown that CMKLR1 protein is reduced in human steatotic liver [15]. However, the liver tissue of only 14 patients was analyzed and gender-related regulation was not determined.

In summary, the present study demonstrates a weak association of hepatic *CMKLR1* expression with features of NASH in male patients.

## 4. Materials and Methods

### 4.1. Study Group

Liver tissues of controls and patients with NAFLD were received and details of the patients are summarized in Table 1. These samples have been introduced in a recent study [26]. Details of the histological scoring which was done as described [27] are summarized in Table 2. The scores were summed up and ranged from 0 to 9. Patients with a score of ≥5 were defined as NASH patients. Alcohol abuse, viral infections and drugs are known to cause liver injury, and therefore, these patients were excluded. Indications for surgery was hepatic metastases of extrahepatic tumours for 70 patients, focal nodular hyperplasia of the liver for nine patients, adenoma for six patients, cholangiocarcinoma for 15 patients, hepatocellular carcinoma for 12 patients and other diseases in 6 patients. Only healthy tissue was used for isolation of RNA. Hypertension, hypercholesterolemia and type 2 diabetes diagnosis had been documented. Serum lipids and glucose were not recorded. Experimental procedures accord to the guidelines of the charitable state controlled foundation Human Tissue and Cell Research and the study was authorized by the local ethical committee of the University of Regensburg (Identification code: 12-101-0048; date: 29 March 2012). The written informed consent was obtained from each patient.

### 4.2. Monitoring of Gene Expression by Real-Time RT-PCR

The LightCycler FastStart DNA Master SYBR Green I kit from Roche (Mannheim, Germany) was used for analyzing the expression of mRNA semi-quantitatively by real-time RT-PCR. Total cellular RNA was reverse transcribed using the Promega Reverse Transcription System (Promega, Madison, WI, USA). The cDNA was used for amplification in glass capillaries (LightCycler, Roche). Oligonucleotides were synthesized by Metabion (Planegg-Martinsried, Germany). Real-time RT-PCR was performed as described and sequencing of the amplified DNA fragments (Geneart, Regensburg, Germany) confirmed the specificities of the PCRs [19]. Serially diluted cDNA was used to create a standard curve for each gene analyzed. The second derivative maximum method was used for quantification with the LightCycler software. Primers to amplify human *CMKLR1* were 5′-ACC TGC ATG GGA AAA TAT CCT-3′ and 5′-GAG GTT GAG TGT GTG GTA GGG-3′. The 18S rRNA was used for normalization and amplified with 5′-GAT TGA TAG CTC TTT CTC GAT TCC-3′ and 5′-CAT CTA AGG GCA TCA CAG ACC-3′.

### 4.3. Statistical Analysis

Data are displayed as box plots and median, lower and upper quartiles and range of the values are shown. The Mann-Whitney *U* Test (SPSS Statistics 21.0 program, IBM, Leibniz Rechenzentrum, München, Germany) was used for comparison of two data sets and Anova followed by a Dunnett post-hoc test was used for comparison of three data sets. ROC analysis and Spearman correlation were done using SPSS Statistics 21.0 program. Youden index was calculated to identify the best cut-off point. A value of *p* < 0.05 was regarded as significant. Distribution of gender and co-morbidities listed in Table 1 was analyzed with the Chi-square test.

## Figures and Tables

**Table 1:** Characteristics of the cohort enrolled in the present study. Data are given as median values and range of the values. Uppercase numbers are shown where data were not available for all of the patients. Significant differences between controls and patients with borderline non-alcoholic steatohepatitis (NASH) are identified by *, between controls and patients with NASH with ^#^ and between patients with borderline NASH and NASH with ^&^.

	Control	Borderline NASH	NASH	*p*-Values
Males/Females	16/17	22/25	24/14	
Age	58 (20–82)	60 (24–84)	66 (33–82)	0.015 ^#^
Body mass index (BMI) kg/m^2^	24.7 (18.3–30.5)	28.0 (22.0–46.0)	28.4 (21.0–57.7)	<0.001 *^,#^
Type 2 Diabetes	0	4	11	0.01 ^#^
Hypertension	7	21	17	
Hypercholesterolemia	0	4	10	
Alanine aminotransferase U/L	21 (8–50) ^32^	35 (17–623) ^36^	32 (10–984) ^35^	<0.001 *^,#^
Aspartate aminotransferase U/L	23 (8–42) ^27^	31 (11–688) ^35^	30 (9–389) ^34^	0.014 * 0.012 ^#^
Alkaline phosphatase U/L	102 (46–203) ^29^	97 (37–444) ^36^	91 (45–826) ^35^	
Bilirubin mg/dL	0.6 (0.19–1.95) ^30^	0.56 (0.19–1.99) ^37^	0.53 (0.20–0.53) ^36^	
Steatosis	0 (0–0)	2 (1–2)	2.5 (1–3)	<0.001 *^,#,&^
Inflammation	0 (0–0)	0 (0–2)	2 (0–3)	0.005 * <0.001 ^#,&^
Fibrosis	0 (0–0)	0 (0–2)	2 (0–4)	0.047 * <0.001 ^#,&^
NASH Score	0 (0–0)	2 (1–4.5)	6 (5–9)	<0.001 *^,#,&^

**Table 2:** Scoring of steatosis, inflammation and fibrosis.

Scores	Description
Steatosis 0	<5% steatosis
Steatosis 1	5%–33% steatosis
Steatosis 2	>33%–66% steatosis
Steatosis 3	>66%
Inflammation 0	No foci/20 × field
Inflammation 1	<2 foci/20 × field
Inflammation 2	2–4 foci/20 × field
Inflammation 3	>4 foci/20 × field
Fibrosis 0	No fibrosis
Fibrosis 1	Zone 3 perisinusoidal/pericellular fibrosis; focally or extensively present
Fibrosis 2	Zone 3 perisinusoidal/pericellular fibrosis with focal or extensive periportal fibrosis
Fibrosis 3	Zone 3 perisinusoidal/pericellular fibrosis and portal fibrosis with focal or extensive bridging fibrosis
Fibrosis 4	Liver cirrhosis

## References

[1] Buechler C., Wanninger J., Neumeier M. (2011). Adiponectin, a key adipokine in obesity related liver diseases. World J. Gastroenterol..

[2] Yeh M.M., Brunt E.M. (2014). Pathological features of fatty liver disease. Gastroenterology.

[3] Friis-Liby I., Aldenborg F., Jerlstad P., Rundstrom K., Bjornsson E. (2004). High prevalence of metabolic complications in patients with non-alcoholic fatty liver disease. Scand. J. Gastroenterol..

[4] Meder W., Wendland M., Busmann A., Kutzleb C., Spodsberg N., John H., Richter R., Schleuder D., Meyer M., Forssmann W.G. (2003). Characterization of human circulating TIG2 as a ligand for the orphan receptor CHEMR23. FEBS Lett..

[5] Yoshimura T., Oppenheim J.J. (2011). Chemokine-like receptor 1 (CMKLR1) and chemokine (C-C motif) receptor-like 2 (CCRL2); two multifunctional receptors with unusual properties. Exp. Cell Res..

[6] Deng Y., Wang H., Lu Y., Liu S., Zhang Q., Huang J., Zhu R., Yang J., Zhang R., Zhang D. (2013). Identification of chemerin as a novel FXR target gene down-regulated in the progression of nonalcoholic steatohepatitis. Endocrinology.

[7] Docke S., Lock J.F., Birkenfeld A.L., Hoppe S., Lieske S., Rieger A., Raschzok N., Sauer I.M., Florian S., Osterhoff M.A. (2013). Elevated hepatic chemerin mrna expression in human non-alcoholic fatty liver disease. Eur. J. Endocrinol..

[8] Krautbauer S., Wanninger J., Eisinger K., Hader Y., Beck M., Kopp A., Schmid A., Weiss T.S., Dorn C., Buechler C. (2013). Chemerin is highly expressed in hepatocytes and is induced in non-alcoholic steatohepatitis liver. Exp. Mol. Pathol..

[9] Rourke J.L., Dranse H.J., Sinal C.J. (2013). Towards an integrative approach to understanding the role of chemerin in human health and disease. Obes. Rev..

[10] Buechler C. (2014). Chemerin in liver diseases. Endocrinol. Metab. Syndr..

[11] Arita M., Ohira T., Sun Y.P., Elangovan S., Chiang N., Serhan C.N. (2007). Resolvin E1 selectively interacts with leukotriene B4 receptor BLT1 and CHEMR23 to regulate inflammation. J. Immunol..

[12] Rouger L., Denis G.R., Luangsay S., Parmentier M. (2013). CHEMR23 knockout mice display mild obesity but no deficit in adipocyte differentiation. J. Endocrinol..

[13] Gruben N., Aparicio Vergara M., Kloosterhuis N.J., van der Molen H., Stoelwinder S., Youssef S., de Bruin A., Delsing D.J., Kuivenhoven J.A., van de Sluis B. (2014). Chemokine-like receptor 1 deficiency does not affect the development of insulin resistance and nonalcoholic fatty liver disease in mice. PLoS ONE.

[14] Ernst M.C., Haidl I.D., Zuniga L.A., Dranse H.J., Rourke J.L., Zabel B.A., Butcher E.C., Sinal C.J. (2012). Disruption of the chemokine-like receptor-1 (*CMKLR1*) gene is associated with reduced adiposity and glucose intolerance. Endocrinology.

[15] Wanninger J., Bauer S., Eisinger K., Weiss T.S., Walter R., Hellerbrand C., Schaffler A., Higuchi A., Walsh K., Buechler C. (2012). Adiponectin upregulates hepatocyte CMKLR1 which is reduced in human fatty liver. Mol. Cell. Endocrinol..

[16] Kukla M., Adamek B., Waluga M., Zalewska-Ziob M., Kasperczyk J., Gabriel A., Mazur W., Sobala-Szczygiel B., Buldak R.J., Zajecki W. (2014). Hepatic chemerin and chemokine-like receptor 1 expression in patients with chronic hepatitis C. BioMed Res. Int..

[17] Pan J.J., Fallon M.B. (2014). Gender and racial differences in nonalcoholic fatty liver disease. World J. Hepatol..

[18] Xin G., Qin S., Wang S., Wang X., Zhang Y., Wang J. (2015). Sex hormone affects the severity of non-alcoholic steatohepatitis through the MYD88-dependent Il-6 signaling pathway. Exp. Biol. Med..

[19] Bauer S., Wanninger J., Schmidhofer S., Weigert J., Neumeier M., Dorn C., Hellerbrand C., Zimara N., Schaffler A., Aslanidis C. (2011). Sterol regulatory element-binding protein 2 (SREBP2) activation after excess triglyceride storage induces chemerin in hypertrophic adipocytes. Endocrinology.

[20] Ernst M.C., Sinal C.J. (2010). Chemerin: At the crossroads of inflammation and obesity. Trends Endocrinol. Metab..

[21] Gonzalez-Periz A., Horrillo R., Ferre N., Gronert K., Dong B., Moran-Salvador E., Titos E., Martinez-Clemente M., Lopez-Parra M., Arroyo V. (2009). Obesity-induced insulin resistance and hepatic steatosis are alleviated by ω-3 fatty acids: A role for resolvins and protectins. FASEB J..

[22] Qiu W., Guo K., Yi L., Gong Y., Huang L., Zhong W. (2014). Resolvin e1 reduces hepatic fibrosis in mice with infection. Exp. Ther. Med..

[23] Pohl R., Rein-Fischboeck L., Meier E.M., Eisinger K., Krautbauer S., Buechler C. (2015). Resolvin E1 and chemerin C15 peptide do not improve rodent non-alcoholic steatohepatitis. Exp. Mol. Pathol..

[24] Serhan C.N. (2007). Resolution phase of inflammation: Novel endogenous anti-inflammatory and proresolving lipid mediators and pathways. Annu. Rev. Immunol..

[25] Sanyal A.J., Abdelmalek M.F., Suzuki A., Cummings O.W., Chojkier M. (2014). No significant effects of ethyl-eicosapentanoic acid on histologic features of nonalcoholic steatohepatitis in a phase 2 trial. Gastroenterology.

[26] Rein-Fischboeck L., Krautbauer S., Eisinger K., Pohl R., Meier E.M., Weiss T.S., Buechler C. (2015). Hepatic scavenger receptor BI is associated with type 2 diabetes but unrelated to human and murine non-alcoholic fatty liver disease. Biochem. Biophys. Res. Commun..

[27] Kleiner D.E., Brunt E.M., Van Natta M., Behling C., Contos M.J., Cummings O.W., Ferrell L.D., Liu Y.C., Torbenson M.S., Unalp-Arida A. (2005). Design and validation of a histological scoring system for nonalcoholic fatty liver disease. Hepatology.

